# Immunomodulatory Effects of Mesenchymal Stromal Cells Revisited in the Context of Inflammatory Cardiomyopathy

**DOI:** 10.1155/2013/353097

**Published:** 2013-06-19

**Authors:** Kapka Miteva, Sophie Van Linthout, Hans-Dieter Volk, Carsten Tschöpe

**Affiliations:** ^1^Berlin-Brandenburg Center for Regenerative Therapies, Charité, University Medicine Berlin, Campus Virchow Clinic, Südstrabe 2, 13353 Berlin, Germany; ^2^Institute of Medical Immunology, Charité, University Medicine Berlin, Campus Virchow Clinic, Südstrabe 2, 13353 Berlin, Germany; ^3^Department of Cardiology and Pneumology, Charité, University Medicine Berlin, Campus Benjamin Franklin, Hindenburgdamm 30, 12203 Berlin, Germany; ^4^DZHK, Deutsches Zentrum für Herz-Kreislauf-Forschung, Berlin, Germany

## Abstract

Myocarditis is a common inflammatory cardiomyopathy, associated with cardiomyocyte apoptosis, which can lead to chronic left ventricular dysfunction. Under conventional heart failure therapy, inflammatory cardiomyopathy typically has a progressive course, indicating a need for alternative therapeutic strategies to improve long-term outcomes. Experimental and clinical studies consistently support the application of cellular transplantation as a strategy to improve myocardial function. Mesenchymal stromal cells (MSCs) mediate distinct paracrine effects supporting endogenous regeneration, but most important are their remarkable immunoregulatory properties. In this review, an overview of current knowledge on immunopathology in myocarditis will be given. Furthermore, current research regarding the immunomodulatory properties of MSCs in the context of myocarditis will be discussed. Finally, the impact of MSC priming by the environment on their functionality and the advantages of systemic administration of MSCs under myocarditis are outlined.

## 1. Introduction

Inflammatory cardiomyopathy (myocarditis), present as an acute and/or a chronic inflammation of the heart, is associated with necrosis and degeneration of cardiomyocytes leading to subsequent cardiac dysfunction [1, 2]. Infections, systemic diseases, drugs, and toxins have been associated with the development of this disease. However, cardiotropic viruses such as Coxsackievirus 3 (CVB3) [3, 4], adenoviruses [5], and parvovirus B19 (B19) [6–8] are considered to be the main cause of inflammatory cardiomyopathy.

The pathology of viral inflammatory cardiomyopathy results from the concomitant work between viral processes of propagation and the host immune responses in attempt to resist and fight against the virus. Both innate and adaptive immune responses are crucial determinants of the severity of myocardial damage, often associated with autoimmune responses against the heart tissue antigens. The overwhelming immune response contributes to the development of chronic myocarditis and dilated cardiomyopathy (DCM), a condition for which the only treatment option at end-stage is heart transplantation [9]. DCM is one of the most common causes of heart failure, contributing to the main mortality rate of cardiomyopathy [10]. Although the application of modern therapy options has led to improved mortality rate, only half of the patients survive for five years [11]. Immunosuppressive and immunomodulating therapy have shown a beneficial effect in chronic, virus-negative inflammatory cardiomyopathy [12, 13], while there is evidence that antiviral therapies [14–17] and antimicrobial agents [18] may have a therapeutic effect in viral or bacterial-induced myocarditis, respectively. Although prominent progress in elucidating the pathophysiological mechanisms of myocarditis and establishment of treatment strategies has been made during the last years, a universal treatment standard is still not available. 

There is accumulating evidence supporting cell therapy as novel treatment option for cardiovascular disorders. MSCs have the advantage over other cells to have a low immunogenicity, allowing unmatched allogenic off-the-shelf use [19]. Furthermore, MSCs do not only have the potential to repair the damaged tissues by secretion of cytoprotective and regeneration supporting factors, but they are also endowed with remarkable immunoregulatory properties. In this review, we will give an overview of current knowledge regarding the immunopathology in myocarditis and discuss current research regarding the immunomodulatory properties of MSCs and the effects of MSCs on immune cells in the context of myocarditis. Further understanding of the mechanisms underlying the interplay between immune cells and MSCs may be helpful in the development of promising approaches to improve cell-based regenerative medicine for cardiovascular diseases and immune therapies.

## 2. Immunopathology in Myocarditis

Myocarditis is caused by a direct cardiac damage due to the invasion and replication of an infectious agent and/or by autoimmune and inflammatory mechanisms, leading to the infiltration of host immune cells, releasing cytokines and autoantibodies against heart antigens which results in focal dying necrotic and apoptotic myofibers [20]. 

However, in most patients severe heart failure does not occur due to the direct viral-induced myocardial injury. In fact, the virus-mediated cardiac damage may go completely unnoticed [21]. Such observations support the idea that inflammation is a leading component and a dominant mechanism in the pathogenesis of inflammatory cardiomyopathy, which is further corroborated by the finding that extensive inflammation in patients with acute myocarditis is an independent predictor of negative outcome [22]. On the other hand, the inflammatory response occurs as a natural defense of the virus-infected heart and plays an important role in pathogen elimination, healing, and tissue repair. However, when the inflammatory response is inadequate and overwhelming, it becomes the cause of a direct injury of cardiac cells and of indirect damage through the action of proinflammatory cytokines and anticardiac autoantibodies, affecting the function of the cardiomyocytes. Therefore, it is not surprising that several preclinical studies have found a beneficial effect of an anti-inflammatory therapy applied during myocarditis [23–26]. 

The evolution of viral myocarditis can be divided in three phases: an acute viremic phase marked by an active replication of live virus within the myocardium. The second phase is a subacute phase characterized by the persistence of the viral genome without detectable viral replication, but with substantial heart infiltration of mononuclear cells. Remodeling in the absence of replicating virus or viral genome defines the chronic phase of viral myocarditis [27]. 

Current understanding of the pathogenesis and the mechanism of myocarditis progression to heart failure, particularly the interaction of inflammatory cells with the cells of the myocardium, is largely based on experimental models using susceptible (A.BY/SnJ and SWR/J) and resistant (C57BL/6J and DBA/1JJ) mouse strains infected with the cardiotropic virus CVB3.

### 2.1. Role of the Innate Immune System in Myocarditis

A broad panel of pattern recognition receptors of the innate immune system and cardiac Toll-like receptors (TLRs) sense viral pathogen-associated patterns [28, 29]. Subsequently, the cells endogenous to the heart—cardiomyocytes, endothelial cells, fibroblasts, and dendritic cells activated via TLRs—respond to the virus infection by releasing cytokines including interleukin (IL)-1*α*, IL-1*β*, IL-6, IL-18, tumor necrosis factor (TNF)-*α*, TNF-*β*, and interferon (IFN)-*γ* [30, 31]. TLR4 expression is prominently increased in endomyocardial biopsies of myocarditis patients and is associated with enteroviral replication and cardiac dysfunction [32].

With respect to inflammatory cardiomyopathy, type I and II IFN activation is known to play an essential cardioprotective role against CVB3 infection. Especially IFN-*γ* expression protects against lethal infections by inducible nitric oxide synthase (iNOS) induction in macrophages and by mediating viral load reduction. Furthermore, experimental and clinical studies have demonstrated that IFN-*β* can lead to elimination of the viral genome and to an improvement of LV function [16, 17, 31, 33, 34].

Natural killer (NK) cells are the first immune cells which infiltrate the myocardium and mediate a cardioprotective effect by limiting the virus replication [35]. However, in spite of the quantitative increase in NK cells subpopulations in the myocardium of patients with cardiomyopathy, functional abnormalities exist in those subpopulations, which are probably related to the pathogenesis of myocarditis [36]. 

In addition to the infiltrated NK cell, various cytokines are produced at that stage, including IL-1*α*, TNF-*α*, IFN-*γ*, and IL-2*α* and cause beneficial and deleterious effects on the myocardial function [37]. Numerous studies have found increased plasma levels of IFN-*γ*, TNF-*α*, IL-6, IL-10, and c-reactive protein in patients with DCM [38–40], clearly indicating chronic immune activation.

In response to those cytokines, NO is released and acts as a modulator of immunological self-defense mechanism, particularly important in the control of virus replication [41]. The classically activated macrophages (M1) promote cardiac inflammation during myocarditis in BALB/c mice via expressing high levels of iNOS [42] and proinflammatory cytokines IL-12, IL-1*β*, and TNF-*α* [25, 43, 44]. In contrast, transfer into susceptible mice of alternatively activated M2 macrophages, producing arginase 1, IL-10, macrophage mannose receptor, and macrophage galactose type C-type lectin [44] has been shown to diminish myocardial inflammation by switching the local cytokine profile from a pro- to anti-inflammatory [25]. 

Dendritic cells (DCs) are the antigen presenting cells with the highest capacity in T-cells priming [45]. Interestingly, a subset of DCs expressing TLR3, as well as higher quantities of IL-10 and the proinflammatory cytokines IL-6 and TNF-*α*, has been shown to be beneficial in the prevention of chronic CVB3 myocarditis [46]. Furthermore, a defect in DCs maturation/activation and DCs MHC class I antigen processing is associated with failure in viral clearance leading to ongoing chronic disease [47].

### 2.2. Role of the Adaptive Immune System in Myocarditis

In the subacute stage, cardiac infiltrating macrophages, DCs, and T cells not only contribute to ongoing cardiac dysfunction [48], but the release of a great variety of cytokines triggers a second wave of immune cells infiltration, including antigen-specific CD4^+^ and CD8^+^T lymphocytes and B cells. 

A pioneer study has defined the prominent role of T cells in the pathogenesis of viral myocarditis, demonstrating that virus infected T-cell-deprived BALB/c mice are protected against lethal infection and exhibit significantly diminished cardiac inflammation [23]. This observation was further confirmed in a more complex study showing that elevated IFN-*γ* and decreased TNF-*α* expression are associated with attenuated myocardial damage in CD4^(−/−)^CD8^(−/−)^ mice [49]. Moreover, those findings indicate that T-cell subsets influence the pathogenesis of myocarditis via modulation of the cytokine expression patterns. Furthermore, cardiac damage in B19-induced myocarditis has been shown to be mediated not only by the primary viral infection and replication within cardiac endothelial cells leading to ongoing vascular injury and subsequent chronic endothelial damage [17], but also via CD8^+^ T cells directed against the myocardium [50]. Furthermore, exacerbated perforin expression in endomyocardial biopsies from patients with acute myocarditis indicates that T lymphocytes might cause a direct cell-mediated cytotoxicity and might injure the myocardium [51].

Patients with myocarditis exhibit an imbalance between helper and cytotoxic T cells [52], providing further evidence that T-cell subpopulations play a great role in the pathogenesis of myocarditis. A recent study in patients with cardiomyopathy revealed a reduced activation of T helper type 1 (Th1) cells producing IFN-*γ* and an impaired production of IL-10, known as a major regulatory cytokine, downregulating an over-enhanced immune response [53]. Those abnormalities in the adaptive immune response could explain to a certain extent the autoimmune features of myocarditis. The cardioprotective role of IL-10 is evident by its ability to reduce proinflammatory cytokines, such as IFN-*γ*, TNF-*α*, and IL-2, in experimental autoimmune myocarditis [54, 55]. Notably, intravenous (i.v.) immunoglobulin treatment of patients with DCM resulted in a significantly increased IL-10 level, which was paralleled by a prominent improvement of left ventricular ejection fraction [56]. 

In the recent years, the concept that the Th1 cytokine IFN-*γ* has regulatory functions and influences the autoimmune reaction has been proven in experimental autoimmune myocarditis, where IFN-*γ* deficiency resulted in the development of exacerbated myocarditis and fatal autoimmune disease [57, 58]. IFN-*γ* is a proven inducer of FoxP3 and the conversion of CD4^+^CD25^−^T cells into CD4^+^ regulatory T cells (Tregs), in both mice and humans [59]. IFN-*γ* is also shown to reduce the progression to chronic myocarditis and DCM via increasing the CD4^+^CD25^+^T regulatory population and reducing fibrosis and pericarditis [60, 61]. 

Moreover, patients with DCM exhibit increased numbers of peripheral Th17 cells associated with autoimmune diseases [62–64] and upregulated levels of the proinflammatory cytokines IL-17, IL-6, and IL-23 [65].

Activated T cells promote clonal expansion and differentiation of B cells, resulting in further injury of cardiomyocytes, enhancement of the local inflammation, and production of circulating antiheart antibodies [66]. It has been widely accepted that viral myocarditis can progress to idiopathic DCM as a consequence of an autoimmune reaction [67, 68]. The percentage of patients with histologically proved myocarditis and serum autoantibodies is ranging from 25 percent to 73 percent [69]. In patients with DCM, autoantibodies have been identified that react with heart mitochondria [70], the adenine nucleotide translocator [71], the muscarinic receptor [72], cardiac troponins [73], myosin heavy chain [74], the second extracellular loop of the beta1-adrenergic receptor [75], and others [76]. 

In summary, evidence from experimental and clinical studies states the complex interactions between distinct immune cells during the different stages of the pathogenesis of inflammatory cardiomyopathy. This underscores the importance of characterizing the stage of the pathogenesis of myocarditis via endomyocardial biopsy analysis [77, 78] to enable an efficient therapy.

## 3. Immunomodulatory Effect of MSCs

Over the past five years, multipotent MSCs have attracted considerable attention, due to their unique immunoregulatory and regeneration supporting properties. Our understanding about the mechanisms of MSC-mediated immunomodulation has been significantly advanced, and MSCs have found a broad application in the fields of inflammatory-mediated disorders and cell therapy. MSCs have a profound effect on components of both innate and adaptive immune response. Numerous reports indicate that the soluble factors released by MSCs induce anergy, apoptosis, Treg cells as well as regulatory (“tolerogenic”) macrophages, and dendritic cells. A particularly important aspect of MSC-mediated immunomodulation is their capacity to migrate to the site of inflammation where the local microenvironment initiates the activation of the MSCs immunomodulatory action, resolving the inflammation.

**Figure 1:**
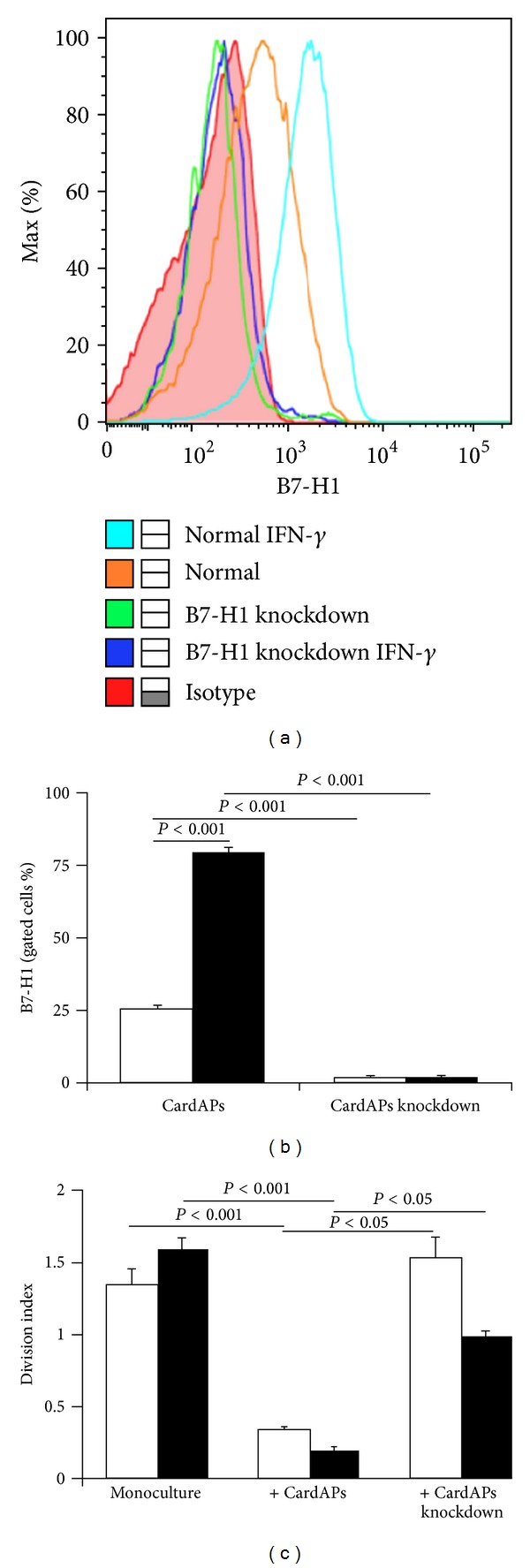
Cardiac-derived cells reduce T-cell proliferation in a cell-contact-dependent manner involving the receptor B7 H-1. (a) Expression of B7-H1 on CardAPs after specific siRNA knockdown. Red coloured histogram represents isotype control, the orange line indicates the expression of B7-H1 on CardAPs under basal conditions. The light blue line represents B7-H1 expression after exogenous stimulation with 5 ng/ml of human IFN-*γ*. The green line shows absence of B7-H1 expression after its specific siRNA knockdown, which even upon IFN-*γ* treatment was not restored (dark blue line). (b) Bar graphs represent the mean ± SEM of B7-H1 positive CardAPs or CardAPs after specific siRNA knockdown of B7-H1, as indicated, depicted as the % of gated CardAPs, under basal culture conditions (open bars) and after 72 h treatment with human IFN-*γ* (closed bars) with *n* = 4/group. (c) The immunosuppressive function of CardAPs is mediated via B7-H1. Spleen MNCs from control (open bars) and CVB3-infected (closed bars) mice were activated and labeled with 10 **μ**M of CFSE to be able to measure cell proliferation, and cultured for 48 h in the absence or presence of CardAPs or CardAPs of which B7-H1 was knocked down, followed by flow cytometry and analysis with FlowJo 8.7. software. Bar graphs represent the division index of spleen MNCs from control and CVB3-infected mice co-cultured for 48 h in the absence or presence of CardAPs or CardAPs of which B7-H1 was knocked down, as indicated, with *n* = 4/group.

MSCs have first been isolated from the bone marrow and identified as colony-forming-unit fibroblasts (CFU fibroblasts) [79]. MSCs are a heterogeneous population of cells that possess stem cell-like characteristics including self-renewal and differentiation capacities *in vitro* [80]. MSCs are predominantly located in perivascular niches and can be found in nearly all tissues. They have been derived from adipose tissue [81], peripheral blood [82], lung [83], placenta, or heart [84]. We recently have isolated and identified novel cardiac-derived cells from endomyocardial biopsies: CardAP cells, previously called CAPs, which show similarities with MSCs [85] and comparably exhibit antiapoptotic [86], proangiogenic, and antifibrotic properties (unpublished data). Among those others, they exhibit prominent immunomodulatory and antiviral features [86, 87]. They reduce the induction of immune cell proliferation, decrease the cardiac levels of TNF-*α*, and not only elevate the proportions of Tregs [86, 87] but also reduce the proportion of apoptotic Tregs in CVB3-infected mice [86]. Similar to MSCs, CardAPs exert their immunomodulatory effects in a paracrine as well as in a cell-contact-dependent manner (Figure 1).

The next subparagraphs are focused at giving an overview of the MSC-mediated immunomodulatory effects. Besides the established impact of MSCs on the adaptive immune response, also their less documented influence on the innate immune response, which plays a leading role in the induction of inflammatory diseases [88], including autoimmune myocarditis [89], will be discussed.

### 3.1. Effect of MSCs on Macrophages

Resident macrophages are known to initiate inflammation in most tissues [90]. Macrophages not only have a great plasticity and can be polarized from classically activated M1 proinflammatory macrophages to alternatively activated M2 anti-inflammatory macrophages, but they also play a key role at the sites of inflammation [91, 92] and determine the severity of myocarditis [25]. Therefore, the ability of MSCs to interact with these cells and to even influence their polarization at the site of inflammation is a particularly important aspect of their immunomodulatory effects. Several studies have demonstrated that MSCs promote the generation of M2 macrophages releasing IL-10, facilitating the resolution of inflammation [93, 94]. MSCs mediate the switch of the macrophages phenotype in a cell-contact-dependent manner via binding of prostaglandin E2 (PGE-2) released by MSCs to PGE-2 receptors on macrophages [93, 95, 96] and/or in a paracrine manner via the release of indoleamine 2,3-dioxygenase (IDO) [94]. Moreover, the proinflammatory microenvironment, containing alloreactivated T cells [97] and IFN-*γ*, TNF, LPS [94, 95], promotes MSC activation via TLR4 and TNFR1 leading to the activation of nuclear factor-*κ*B (NF-*κ*B) signaling and subsequent expression of cyclooxygenase 2 and IDO in MSCs, which further induces macrophages polarization to the M2 type [97]. Importantly, the MSC-mediated protective effect has been shown to be abrogated after macrophage depletion or anti-IL-10 neutralizing antibodies application, furthermore highlighting the relevance of MSC-mediated reprogramming of macrophages, preventing excessive inflammatory responses and supporting tissue regeneration [95]. The ability of MSCs to induce M2 macrophages is of great relevance for resolving the inflammation and for the prevention of the development of autoimmune myocarditis not only due to the release of the anti-inflammatory and cardioprotective cytokine IL-10 [54, 95] but also due to the critical role of M2 macrophages in the induction of Treg cells in myocarditis [55]. In addition, the anti-inflammatory protein TNF-stimulated gene- (TSG-) 6 secreted by activated MSCs has been shown to attenuate peritonitis by decreasing TLR2/NF-*κ*B signaling in resident macrophages [98]. Another paracrine factor involved in the interaction of MSCs with macrophages is the IL-1 receptor antagonist, which is released by MSCs and abrogates the secretion of TNF by activated macrophages [99].

### 3.2. Effect of MSCs on Natural Killer Cells

MSCs inhibit not only the cytokine-induced proliferation of freshly isolated NK but also their cytotoxic function [100], cytokine production, granzyme B release, and activating receptors expression [101–105]. They inhibit the function of NK cells mainly in a contact-dependent manner and to a lesser extent by the release of paracrine factors such as IDO and TGF-*β* [105–107]. Since NK cells are important effector cells with cardioprotective properties, limiting virus replication [35] through the release of IFN-*γ* [108], an MSC-mediated repressive effect on NK cells at an early stage of infection when NK cells play an indispensable role in the inhibition of the viral replication [35] could favor viral propagation and exacerbation of autoimmune myocarditis. This hypothesis is supported by the finding that functional abnormalities of NK [36] or NK cells depletion [109] contributes to the pathogenesis of myocarditis. On the other hand, overactivation and/or long-term presence of NK cells may abrogate the myocardial damage due to the excessive release of cytotoxic molecules such as perforin within the myocardium, known to contribute to myocardial necrosis and lymphocyte infiltration [110]. Therefore, the MSC-mediated inhibitory effect on NK function in the case of overactivation and NK cells persistence in the myocardium could be beneficial for resolving the inflammation and diminishing the myocardial damage.

Overall, the consequence of the MSC-mediated suppressive effect on NK function strongly depends on the stage of myocarditis. Via their suppressive effect, they could either support viral propagation in the viremic phase or could be beneficial reducing myocardial damage, decreasing the inflammation in the subacute phase.

### 3.3. Effect of MSCs on Dendritic Cells

MSCs have a profound effect on DCs which perform antigen acquisition, processing, and antigen presentation to naive T cells and are therefore of crucial importance for the direction of the development of the adaptive immune responses. Importantly, MSCs can influence the phenotype of the DCs, diminishing the percentage of DCs with a conventional phenotype, while promoting the plasmacytoid DCs, directing hereby the adaptive immune response towards the Th2 type [111]. Importantly, this MSC-mediated induction of plasmacytoid DCs and subsequent development of a Th2 response is of great relevance in the context of myocarditis due to the protective effect of the Th2 polarization preventing the development of autoimmune heart disease [112, 113]. Moreover, the MSC-mediated induction of plasmacytoid DCs which are a source of type-I IFN further exerts direct antiviral effects [113]. In addition, plasmacytoid DCs upregulate the release of the anti-inflammatory and cardioprotective cytokine IL-10 in the presence of MSCs [107], which could further protect against an overwhelming immune response and cardiac damage. Besides influencing the DC phenotype, MSCs inhibit DCs differentiation, maturation, and impair the antigen-presenting function of the DCs [114–116]. Moreover, several studies have demonstrated that MSCs interfere with the capacity of DC to migrate to the local lymph node [117], which further significantly affects the ability of DCs to prime T cells in the draining lymph. It will be of great interest to define the effect of MSC application on the migration and homing capacity of DCs in the context of myocarditis.

### 3.4. Effect of MSCs on the T-Cells Balance (Th1/Th2/Th17/Tregs)

T cells are a major player in the adaptive immune response. They are activated through the TCR receptor proliferate and perform numerous effector functions, including cytokines release and inducing cytotoxicity in case of CD8^+^ T cells (CTL), which all contribute to cardiac damage [118]. Numerous publications have shown that MSCs successfully inhibit T-cell proliferation *in vitro* and *in vivo* [119–125]. In addition, MSCs are able to induce T-cells apoptosis, and a very recent study has revealed the exact mechanism demonstrating that after systemic infusion of MSCs, T cells underwent apoptosis via the FAS ligand-dependent FAS pathway [126]. In agreement with this finding, we have shown that the application of MSCs as well as of the MSCs-like CardAPs results in MSCs/CardAPs-induced apoptosis of CD4^+^ and CD8^+^ T cells in a model of CVB3-induced inflammatory cardiomyopathy [86, 127]. 

Moreover, MSCs exert a systemic suppressive effect by mediating a polarization of the host immune response towards a Th2 profile, independently from Tregs induction [128], which precludes the development of an autoimmune heart disease [112]. Furthermore, MSCs not only promote a dominant Th2 phenotype but also decrease the proinflammatory cytokines IL-17 and IFN-*γ* and increase the levels of IL-4, IL-10, and TGF-*β* in models of autoimmune disease [129, 130]. Interestingly, we have found that in a model of viral-induced myocarditis, application of MSCs [127] and CardAPs [86] promotes the upregulation of IFN-*γ*, known to reduce CVB3 replication [131]. Additionally, CVB3-infected mice receiving either MSCs or CardAPs had elevated cardiac mRNA expression levels of IL-10, and MSCs-treated mice had significantly higher proportion of IL-10-producing CD4^+^ and CD8^+^ T cells [127], which leads to diminished severity of the disease. Importantly, both IL-10 [132] and IFN-*γ* [133] are crucial in the functionality of Tregs. Another cytokine involved in Treg cells induction is TGF-*β*, which is released during the process of macrophage phagocytosis of the apoptotic T cell, which had gone to apoptosis after MSCs application [126].

In addition, MSCs inhibit the formation of CTL but preserve the activated CTL with a restricted killing of virally infected cells [102]. This balanced effect of MSCs allowing a precise killing of virally infected cells is of great benefit restricting the CTL activity only on virally infected cells and preventing the damage of healthy cardiomyocytes.

In several immune-mediated diseases, the transfer of MSCs has been accompanied by a decrease in Th17 cell activity, leading to a significant disease improvement [128, 134]. Moreover, MSCs not only efficiently suppress the production of the proinflammatory cytokines IL-17 and IL-22 by Th17 cells but also could reprogram Th17 cells into IL-10^+^FoxP3^+^ Treg cells [135]. In addition, human and mouse MSCs prevented the *de novo *generation of Th17 cells from naive T cells [136].

Tregs play an essential role in the regulation of the immune responses and in the prevention of autoimmune disease. MSCs-induced tolerance is often associated with a promotion of Tregs [137–139]. The importance of Treg cells for the immunomodulatory effect of MSCs is evident from studies showing that a depletion of Tregs abrogates the anti-inflammatory effect mediated by MSCs [139, 140]. As previously discussed, MSCs favour the generation of Tregs, and this corresponds with a decrease in Th1, Th2, and Th17 lymphocytes [126, 129, 135, 141]. It has been shown that MSCs could promote Treg cells in a contact-dependent manner [142] as well as by releasing PGE-2 and TGF-*β*1 [143]. MSC-mediated expansion or induction of Treg cells has been associated with beneficial effects in a number of alloreactive, autoimmune, and allergic diseases including organ transplantation [137, 138, 144, 145], type 1 diabetes, [130, 146] and inflammatory bowel disease [126, 147] as well as acute CVB3-induced myocarditis [127]. The protection afforded by MSCs in these models is mediated through multiple mechanisms, which in the end lead to the generation of functionally active Tregs and subsequent establishment of an immune tolerance. Although the number of transplanted MSCs, which initially suppress the pathogenic Th2 phenotype, decreases over time, MSC-induced Tregs expand and persist and could be the key cells establishing a sustained immune control long after MSCs have disappeared [148].

Both cell-cell contact and soluble factors have been implicated in MSC-mediated immunosuppression. It has been shown that IFN-*γ* acts directly on MSCs and leads to the upregulation of B7 homolog 1 (B7-H1), known to function by interaction with its cognate receptor on target cells, a process mediated by cell-cell contact and facilitating MSCs-induced inhibition of lymphocyte proliferation [149]. In line with this notion, we have found that CardAPs similarly to MSCs upregulate the expression of B7-H1 upon IFN-*γ* stimulation (Figure 1(a)), and B7-H1 knockdown of CardAPs abolished the antiproliferative effects on activated splenocytes (Figures 1(a) and 1(b)). The described finding suggests that the immunomodulatory effects of CardAPs are at least partly mediated in a contact-dependent manner via B7-H1 interaction.

Several soluble immunosuppressive factors are either produced constitutively by MSCs or released by MSCs upon activation and contribute to the immune-regulation. For example IDO is only released by MSCs after IFN-*γ* stimulation [150, 151]. However, the soluble factors TGF-*β*1, hepatocyte growth factor [152], IL-10, PGE-2, haem-oxygenase-1, and IL-6 are constitutively produced by MSCs [153, 154]. 

Human leukocyte antigen-G5, which is also constitutively produced by MSCs, suppresses T-cell proliferation, as well as NK cell and T-cell cytotoxicity, while promoting the generation of Tregs [142] is another relevant factor for the MSC-mediated regulation of the immune response. Furthermore, MSCs have been shown to express the anti-inflammatory protein TSG-6 upon engraftment in the lung and subsequent activation after i.v. injection in a myocardial infarction model. This MSC-mediated release of TSG-6 suppressed the excessive inflammatory response in the heart, resulting in improved cardiac function and diminished scarring of the left ventricle [155]. 

None of the previous paracrine factors released by MSCs seems to have an exclusive role in the MSC-mediated immune regulation, which is probably a result of a combination of mechanisms. 

### 3.5. Effect of MSCs on B Cells

B cells are central contributors to the pathogenesis of immune-related disorders [156] and particularly of myocarditis [157], due to their ability to produce antibodies as well as their role in antigen presentation, modulation of the spleen architecture, and Th1/Th2 polarization of T cells [156]. On the other hand, a distinct regulatory B-cells (Bregs) subset with suppressive functions, including inhibition of Th1-, Th17-, or induction of Th2-mediated responses, conversion of effector T cells into IL-10-producing T cells, and maintenance of FoxP3 expression in the Treg pool, has been identified and studied [158, 159]. Knowing that MSCs promote the generation of Tregs [137–139], it will be of interest to investigate whether MSCs can also promote Bregs, which could exert a great variety of immunomodulatory effects and consequently could also be beneficial in resolving the inflammation in myocarditis. 

The immunomodulatory properties of MSCs also include a direct effect on B-cell activities. MSCs inhibit B-cell proliferation and terminal differentiation [160] to immunoglobulin IgM-, IgG-, and IgA-antibody secreting cells and B-cell chemotaxis [161]. The finding that MSCs injected *in vivo* home to lymph nodes and the spleen where they cluster around T cells [162] supports the hypothesis that B cells may also be targeted by MSCs in the T-cell area of secondary lymphoid organs. Subsequently, this effect could be beneficial in the subacute stage of myocarditis, preventing the induction of a second wave of immune cell infiltration, including antigen-specific CD4^+^ and CD8^+^ T lymphocytes. 

## 4. Priming of MSCs

The view that MSCs require priming or activation in order to fulfill their immunosuppressive function is already a well established concept. It has been repeatedly demonstrated that IFN-*γ*, TNF-*α*, or IL-1*β* are the essential factors necessary for MSCs activation and the subsequent induction of immunomodulatory effects [150, 163, 164]. In agreement with those findings, our research revealed that MSCs require IFN-*γ* priming to exert their antiapoptotic, antioxidative, and antiviral features, and the supplementation of IFN-*γ* to CVB3-infected MSCs increased nitric oxide (NO) production. This suggests that in the context of CVB3 and upon activation with IFN-*γ*, MSCs produce NO via which they exert their antiapoptotic and antioxidative effects, leading to a decrease in viral progeny release and viral production [127]. Importantly, it has been shown that MSCs derived from IFN-*γ* receptor deficient mice have lost their immunosuppressive activity, which highlights the exclusive role of IFN-*γ* in the immunosuppressive function of MSCs [163]. Moreover, we found that CardAPs require priming via IFN-*γ* in order to exert their antiapoptotic and immunomodulatory features too, and upon IFN-*γ* supplementation, CVB3-infected CardAPs raised significantly the level of the anti-inflammatory cytokine IL-10 [86]. The importance of IFN-*γ* priming for the functionality of MSCs/CardAPs on the one hand and the increased cardiac IFN-*γ* levels in acute CVB3-induced myocarditis on the other hand [127] support the hypothesis that MSCs/CardAPs will exert their protective effects in the cardiac inflammatory environment of acute CVB3-induced myocarditis. Furthermore, the induction of cardiac IFN-*γ* mRNA expression upon MSCs/CardAPs application suggests an autonomous self-regulatory feedback loop ensuring the activated state of MSCs/CardAPs [86, 127].

Interestingly, MSCs derived from different sources have been reported to express TLR 1, 2, 3, 4, 5 [165–169]. The inflammatory environment has a great influence on the TLRs expression pattern in MSCs, and the proinflammatory cytokines IFN-*α*, IFN-*γ*, TNF-*α*, and IL1-*β* promote TLR 2, 3, and 4 expression while downregulate TLR 6 expression [170]. Moreover, TLR signaling in MSCs has also been implicated in the priming of MSCs. For example, TLR 3 and TLR 4 activation of MSCs provoked an MSC-mediated immunosuppression via IDO induction and IFN-*β* and protein kinase R signaling [171], while TLR 2 activation of MSCs resulted in the upregulation of the immune-suppressive protein galectin-3 [172]. By contrast, another study demonstrated that MSC activation through TLR 3 and 4 resulted in the loss of the immunomodulatory effects of MSCs via downregulation of Jagged-1 [173]. Overall, TLR signaling has the capacity to convert MSCs to cells with a proinflammatory and antigen-presenting phenotype, releasing proinflammatory cytokines and chemokines, which further promote inflammation [174]. In line with the previous observations, Waterman et al. proposed the idea that the induction of proinflammatory (MSC1) or anti-inflammatory (MSC2) phenotypes of MSCs depends on the ligand concentration, the timing, and the kinetics of activation [175]. For example, TLR 4 activation results in the upregulation of the classical proinflammatory cytokines IL-6, IL-8, or TGF-*β* (MSC1 phenotype) and activation of T lymphocytes, while TLR 3 priming results in the production of anti-inflammatory molecules like IL-4, IDO, or PGE2 (MSC2 phenotype) and inhibition of lymphocyte proliferation [175].

Further investigation is required on the effects of TLR activation on MSCs. However, these findings indicate that MSCs are cells with a great plasticity, which is modulated by the factors of the environment, and point out that MSCs may have the capacity to promote pathogen clearance or immune suppression as well as inflammation.

## 5. Route of Application

Whereas the low cardiac engraftment of MSCs after i.v. injection compared to intracoronary or endocardial application is well established [176], only recent studies have indicated the potential benefit of extracardiac targeting of MSCs for the cardiac outcome. Lee et al. [155] demonstrated that MSCs upon entrapment in the lung after i.v. injection expressed the anti-inflammatory protein TSG-6 and improved myocardial infarction via—at least in part—the release of this anti-inflammatory protein. This followed from the finding that MSCs transduced with TSG-6 siRNA did not exert these protective effects, whereas i.v. application of recombinant TSG-6 also did. The same authors [177] further demonstrated in a chemical injury model of the cornea that systemic administration of MSCs reduced inflammatory damage to the cornea without engraftment, underscoring their mode of action at a distance. Kavanagh and Mahon [140] provided evidence that i.v. MSC application prevents allergic airway inflammation by inducing murine Tregs following from the finding that under the use of a moderate dose of cyclophosphamide to differentially ablate Treg responses, the major beneficial effect of MSC therapy was lost. With respect to CVB3-induced myocarditis, we demonstrated that both i.v. application of MSCs [127] and of CardAPs [86] resulted in an induction of Tregs, whose protective effects in myocarditis have been consistently demonstrated [60, 61] and in a raise of splenic apoptotic CD4^+^ and CD8^+^ T cells. With the spleen being a reservoir of monocytes, which are recruited to the inflammatory heart [178], we suggest that these MSC/CardAPs-mediated immunomodulatory effects in the spleen contributed to the cardioprotective effects of MSCs/CardAPs in inflammatory cardiomyopathy. This hypothesis is supported by the findings that (i) splenectomy improves the myocardial infarction outcome [179], (ii) the use of an antibody against T cells reduces the cardiac damage in myocarditis [180], and (iii) our recent observation that splenocytes isolated from CVB3-infected mice injected with MSCs induce less collagen production in fibroblasts compared to splenocytes from CVB3 mice [127].

**Figure 2:**
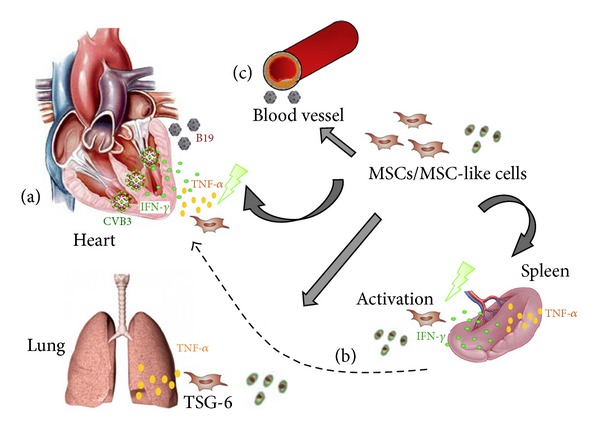
Hypothetical scheme representing how the cardiac outcome in inflammatory cardiomyopathy may be influenced after i.v. injection of MSCs or the MSC-like CardAPs. Via (a) direct cardioprotective effects, via (b) extracardiac immunomodulatory effects involving MSCs/CardAPs-mediated immunomodulation in the spleen and/or other secondary lymphoid organs, and the induction of tumor necrosis factor-stimulated gene- (TSG-) 6 expression by human MSCs/CardAPs upon engraftment, for example, in the lung, and via (c) systemic endothelial-protective effects. The contribution of (a), (b), or (c) on the final cardiac outcome is expected to depend on the kind of inflammatory cardiomyopathy, be it viral- (CVB3 or B19-induced) or nonviral- induced.

The limited number of cardiac engrafted MSCs after i.v., intracoronary, and endocardial application [127, 155, 176], their limited residence time [155], and their action into their near proximity upon engraftment [163] suggest that the improvement in cardiac function after application cannot be solely due to the cardiac engrafted MSCs and further supports the significance of the beneficial effects of extracardiac targeting, be it by the induction of paracrine factors and/or of Tregs.  Figure 2 represents a hypothetical scheme of how MSCs or the MSC-like CardAPs can improve the cardiac outcome after i.v. injection in inflammatory cardiomyopathy and addresses the direct cardiac-protective as well as systemic protective effects of MSCs/CardAPs following extra-cardiac targeting.

The capacity of MSCs to home to the site of injury/inflammation [181, 182] is another argument in favor of the i.v. application route. In agreement, we demonstrated that the cardiac homing of endogenous MSCs in patients depends on the grade of inflammation in the heart [182]. Furthermore, the migration of MSCs from the circulation to damaged tissue leading to a therapeutic effect has already been demonstrated [183–185]. We foresee that especially a cellular therapy option for B19-induced inflammatory cardiomyopathy, which is associated with a systemic endothelial dysfunction and endothelial damage [6, 17], could profit from a systemic MSC application ensuring an endothelial engraftment.

Finally, the noninvasiveness of the i.v. administration route, avoiding all risks of transendothelial and intracoronary injections, and the ability of performing reinjections make the i.v. application of MSCs an attractive therapeutic strategy. Very recent unpublished data suggest that intramuscular injection could induce systemic immunoregulatory properties protecting mice from lethal irradiation or supporting revascularization in critical limb ischemia (even if injected distant from the targeted limb). This opens a new avenue of application.

An overview of (dis)advantages of i.v., intracoronary, and endocardial MSC application for the treatment of inflammatory cardiomyopathy is given in Table 1.

## 6. Conclusion and Perspectives

In summary, it seems that MSCs do not simply inhibit cells of the immune system but rather modulate the function of a great variety of immune cells to establish a fine balance between pathogen elimination and repair processes. However, it has to be noted that many effects cannot be reproduced with all MSC types and all laboratories. The reasons for discrepancies might be the poor characterization of the MSCs used (source, species, 2D versus 3D expansion), the various age of donor (e.g. old bone marrow donors during hip replacement operation versus cord blood), and poor culture conditions (MSCs express high metabolic activities that can result *in vitro* in simple metabolic effects). Moreover, the mode of action of many effects is poorly understood. The coordinating signals involved in this balance remain to be elucidated, and the systemic effects of MSCs on the various immune cell types need to be extensively analyzed, particularly in view of the establishment of a physiological immune homeostasis in inflammatory cardiomyopathy. Since the cell dose affects the amount of induced Tregs [140] as well as of lung engrafted MSCs [155] and therefore subsequent extracardiac-mediated protective effects, further studies are required clarifying the optimal MSC dose in experimental models of inflammatory cardiomyopathy. Since so far MSCs have been shown not to be able to reduce the viral load in acute CVB3-induced myocarditis [127], despite their direct antiviral effects demonstrated *in vitro *[187], further studies are necessary evaluating the effect of MSC application in chronic CVB3-induced myocarditis. This investigation is also recommended for CardAPs, which did reduce the viral load in acute CVB3-induced myocarditis [86], since only in this setting it will be clear whether MSC and/or CardAPs application is not associated with viral rebound and can be used for the treatment of viral-induced inflammatory cardiomyopathy. With respect to chronic (non)viral-induced inflammatory cardiomyopathy, the impact of the time point of MSC/CardAPs application during the pathogenesis of the disorder on the cardiac outcome needs further investigation. Especially in the case of chronic inflammatory cardiomyopathy, where depending on the stage of injection, inflammation could be less prominent, and consequently the activation of MSCs could be decreased, further clarifications are required on the impact of preconditioning of the cells or of cotreatments.

Finally, the responsiveness of the patient towards the cell therapy should be estimated by analyzing the expression of chemokines and inflammation markers in the heart via the isolation of endomyocardial biopsies [188] and subsequent gene expression analysis.

These insights are still required enabling an optimal clinical application of MSCs for the treatment of inflammatory cardiomyopathy.

## Figures and Tables

**Table 1:** Advantages and disadvantages of different cell administration routes for the treatment of inflammatory cardiomyopathy. As long as no studies are performed directly comparing the effectiveness of intravenous versus endocardial and intracoronary application in inflammatory cardiomyopathy, one cannot conclude that extracardiac targeting of MSCs is superior to high cardiac engraftment.

Application	Advantages	Disadvantages
Intravenous	Noninvasiveness Ability to perform reinjections [186] Homing to the site of injury and inflammation [181, 182] Extracardiac targeting [155] Systemic immunomodulatory effects [86, 127]	Low cardiac engraftment [127, 155, 176]? Limited cardiac residence time?

Endocardial	Higher cardiac engraftment [176]?	Invasiveness of application Inability to perform reinjections Limited cardiac residence time [155]

Intracoronary	Higher cardiac engraftment [176]?	Invasiveness of application Inability to perform reinjections Limited cardiac residence time [155]

## Abbreviations

Breg: Regulatory B cells
B7-H1: B7 homolog 1
B19: Parvovirus B19
CardAPs: Cardiac-derived proliferating cells
CTL: Cytotoxicity CD8^+^ T cells
CVB: Coxsackievirus
DCM: Dilated cardiomyopathy
DCs: Dendritic cells
IDO: Indoleamine 2,3-dioxygenase
IFN: Interferon
Ig: Immunoglobulin
iNOS: Inducible nitric oxide synthase
IL: Interleukin
i.v.: Intravenous
LV: Left ventricle
MSC: Mesenchymal stromal cell
M1: Classically activated macrophages
M2: Alternatively activated macrophages
NF-*κ*B: Nuclear factor *κ*B
NK: Natural killer
PGE2: Prostaglandin E2
Th: T helper type
TLR: Toll-like receptor
TNF: Tumor necrosis factor
TGF: Transforming growth factor
Tregs: Regulatory T cells
TSG-6: Tumor necrosis factor-stimulated gene-6.
